# Physiological Characteristic Changes and Transcriptome Analysis of Maize (*Zea mays L.*) Roots under Drought Stress

**DOI:** 10.1155/2024/5681174

**Published:** 2024-01-17

**Authors:** Chenglin Zou, Hua Tan, Kaijian Huang, Ruining Zhai, Meng Yang, Aihua Huang, Xinxing Wei, Runxiu Mo, Faqian Xiong

**Affiliations:** ^1^Maize Research Institute, Guangxi Academy of Agricultural Sciences, Nanning 530007, Guangxi, China; ^2^Cash Crops Research Institute, Guangxi Academy of Agricultural Sciences, Nanning 530007, Guangxi, China

## Abstract

Water deficit is a key limiting factor for limiting yield in maize (*Zea mays L.*). It is crucial to elucidate the molecular regulatory networks of stress tolerance for genetic enhancement of drought tolerance. The mechanism of drought tolerance of maize was explored by comparing physiological and transcriptomic data under normal conditions and drought treatment at polyethylene glycol- (PEG-) induced drought stress (5%, 10%, 15%, and 20%) in the root during the seedling stage. The content of saccharide, SOD, CAT, and MDA showed an upward trend, proteins showed a downward trend, and the levels of POD first showed an upward trend and then decreased. Compared with the control group, a total of 597, 2748, 6588, and 5410 differentially expressed genes were found at 5%, 10%, 15%, and 20% PEG, respectively, and 354 common DEGs were identified in these comparisons. Some differentially expressed genes were remarkably enriched in the MAPK signaling pathway and plant hormone signal transduction. The 50 transcription factors (TFs) divided into 15 categories were screened from the 354 common DEGs during drought stress. Auxin response factor 10 (ARF10), auxin-responsive protein IAA9 (IAA9), auxin response factor 14 (ARF14), auxin-responsive protein IAA1 (IAA1), auxin-responsive protein IAA27 (IAA27), and 1 ethylene response sensor 2 (ERS2) were upregulated. The two TFs, including bHLH 35 and bHLH 96, involved in the MAPK signal pathway and plant hormones pathway, are significantly upregulated in 5%, 10%, 15%, and 20% PEG stress groups. The present study provides greater insight into the fundamental transcriptome reprogramming of grain crops under drought.

## 1. Introduction

Drought, or water deficit, is one of the major environmental constraints to terrestrial plants, which limits agricultural production worldwide and generates a prodigious threat to our food security. Many physiological responses can be triggered by drought stress, such as wilting, growth arrest, alterations in metabolism, closure of stomatal cells, and even death under severe conditions in plants [1, 2]. Owing to water deficit, CO_2_ assimilation in photosynthesis evidently decreases and impairs sugar biosynthesis. Gene expression under drought is affected by phytohormones, especially abscisic acid (ABA) and auxin [3].

Meanwhile, drought stress leads to oxidative stress by inducing the accumulation of toxic reactive oxygen species (ROS) and inducing the antioxidant system [4]. Phytohormones have a significant influence on the expression of abscisic acid (ABA) and auxin genes owing to drought stress [5]. These changes can further reduce crop yields. It has been reported that rice (*Oryza sativa L.*) suffered a drastic water deficiency, which resulted in yield reduction range of 18-60% [6, 7]. Meanwhile, drought stress leads to a 10-50% reduction in wheat [8]. The production of maize (*Zea mays L.*) and barley (*Hordeum vulgare L.*) decreased by 1-76% and 73-87% owing to drought stress, respectively [9]. Similar reports have also been found in leguminous crops, including pigeon peas (*Cajanus cajan* (*Linn*.) *Millsp*.), chickpeas (*Cicer arietinum Linn.*), and rape (*Brassica napus L.*), which are planted on dry land, and the yield has declined seriously due to water shortage [10].

Maize is not only of importance throughout the world as a source of food, feed, and various important industrial products but also is a model genetic organism with great genetic diversity. Although it was first domesticated in Mexico, varieties of maize are widely found on the continents, and maize has become the most extensively cultivated cereal crop, followed by wheat and rice [11]. However, the production of maize in many developing countries is severely limited by a range of abiotic or biotic stresses, such as drought stress. Previous research indicated that maize is highly sensitive to drought, and it has shown that drought stress can reduce maize yield by 10–76% [12], especially moisture deficit at the seedling stage can affect the entire growth cycle, thus affecting the early adaptation ability of plants. Therefore, it is important to elucidate the mechanism of how maize responds to drought stress during seedling stage.

Furthermore, the regulation of stress-responsive genes plays an important role in response to drought stress, which is regulated by various transcription factors such as the WRKY family, the AP2/ERF superfamily, and the bHLH family [13]. Mitogen-activated protein kinase (MAPK) cascade was potentially significant signaling pathways involved in transducing external stimuli to the nucleus for appropriate adjustment of cellular responses under stress [14]. MAPK signaling pathway has been confirmed to play crucial roles in the response to multiple abiotic stresses, such as drought stress. Under stress conditions, the phosphorylation of target genes is regulated by MAPK, which controls various transcription factors involved in abiotic stress tolerance [15]. It is important to explore the molecular regulation of drought stress in maize. Meanwhile, a total of 11 drought stress-associated DEGs were annotated as late embryogenesis abundant protein genes, which were largely expressed at polyethylene glycol-simulated drought stress [16].

Through transcriptomic analysis of Eruca vesicaria subs, sativa lines found 51 genes which were significantly upregulated under polyethylene glycol-simulated drought stress, including those for ethylene-responsive transcription factors, WRKY and bHLH transcription factors, calmodulin-binding transcription activator, cysteine-rich receptor-like protein kinase, mitogen-activated protein kinase, allene oxide cyclase, aquaporin, C-5 sterol desaturase, trehalose-phosphate phosphatase, and galactinol synthase 4 [17].

Meanwhile, NGS-based RNA-Seq has been applied as a comprehensive high-throughput method to reveal regulatory networks in various species [10]. In this study, we analyzed genes that were differentially expressed, comparing transcriptomic data under normal conditions and drought treatment at polyethylene glycol- (PEG-) induced drought stress (5%, 10%, 15%, and 20%). Analyzing these transcriptomic data reveals the early dynamic molecular regulation of drought stress in maize and indicates the key genes responsible for drought tolerance, which is important for the drought tolerance of drought response in maize roots.

## 2. Materials and Methods

### 2.1. Plant Materials

#### 2.1.1. Sample Preparation

**Figure 1:**
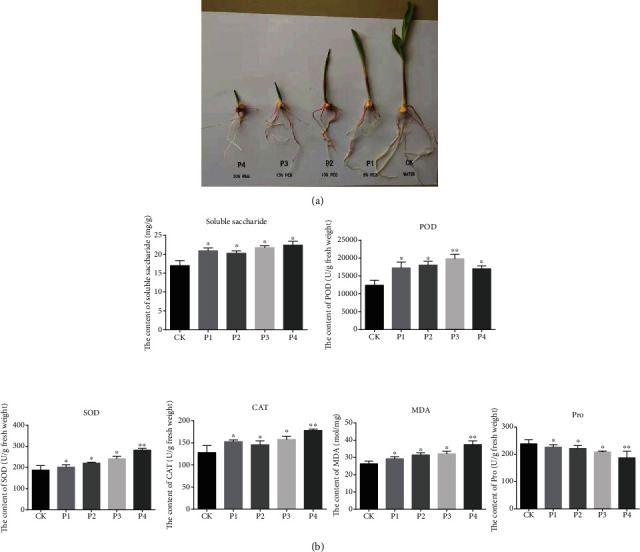
The effects of polyethylene glycol- (PEG-) induced drought stress in maize. (a) Control: seeds at control media (without PEG). P1: 5% PEG-induced drought on root developments. P2: 10% PEG-induced drought on root developments. P3: 15% PEG-induced drought on root developments. P4: 20% PEG-induced drought on root developments. (b) The effect of PEG treatment on the content of soluble saccharide, POD, SOD, CAT, MDA, and protein. The asterisk represents the standard deviation. Bar graph with different asterisk indicates significant difference between the mean values (*p* ≤ 0.05).

Seeds of maize inbred line Q901 (the parent of the maize variety Zhaoyu 215) are provided by Maize Research Institute, Guangxi Academy of Agricultural Sciences. The seeds with full particles, consistent size, and no damage were selected. The seeds were soaked in 75% alcohol for 3 minutes for disinfection, and distilled water from filter paper of germinating box was 121 minutes, and auto sterilization was performed for 30 min. With water as a control treatment (CK), polyethylene glycol- (PEG-) 6000 solution with different concentrations of 5% (P1), 10% (P2), 15% (P3), and 20% (P4) was used to simulate drought stress. In each germinating box place 30 seeds, 20 mL peG-6000 solution of different concentrations was added in the germinating boxes, respectively. The germinating boxes were put into the artificial climate box to perform a germination experiment. The constant temperature was 25°C, and the relative humidity was 80. After cultivating for 8 days, we removed the seedlings from the germinating box (Figure 1(a)). The radicles of germinated seeds were cut, put in an airtight bag, and stored at -80 for later use. Three replicates were collected for transcriptome analysis and physiological index measurement.

The maize rootstocks were sampled to estimate the physiological indexing and MDA (malondialdehyde) levels. Each sample was 0.1 g fresh tissue in 1 mL precooled PBS buffer. After centrifugation at 10,000 g for 10 min at 4°C, adduct formation was measured using Thermo Scientific Multiskan FC (Shanghai, China) at 405 nm on a spectrophotometer (Thermo Scientific Multiskan FC, Shanghai, China). Protein contents were determined using an Enhanced BCA Protein Assay Kit (Beyotime, Shanghai, China). The activities of antioxidant enzymes, including catalase (CAT), superoxide dismutase (SOD), and peroxidase (POD), were measured as described previously. Three biological replications determined all the above physiological indicators. The samples were treated according to previous research [18].

#### 2.1.2. RNA Extraction, cDNA Library Construction, and RNA-Seq

Total RNAs were extracted using TRIzol reagent (Invitrogen, Carlsbad, CA, USA) following the manufacturer's procedure. The quantities and qualities of RNA with RIN values greater than 7 were assessed using an Agilent Bioanalyzer, and the concentration was measured to be 327 ng/*μ*L. The integrity of RNA was assessed by agarose gel electrophoresis. After purifying approximately 10 ng of total RNA with poly-T oligo-attached magnetic beads, it was lysed into smaller fragments using a fragmentation buffer (Solarbio, Beijing, China). Subsequently, reverse transcriptase and random hexamer primer were used to transcribe cleaved RNA fragments into first-strand cDNA fragments. The RNA-Seq sample preparation kit (Illumina, San Diego, CA, USA) was used to construct the cDNA library. An Illumina Hiseq4000 (LC Sciences, San Diego, CA, USA) was used to perform paired-end sequencing.

#### 2.1.3. Quality Control, DEG Analysis, and Gene Ontology and Gene Pathway Enrichment Analysis

Acquired raw data was further processed including removal of adaptor and low-quality sequence reads for obtaining high-quality data [19]. The TopHat package [20] was employed to compare the valid dates with the maize reference genome. Then, Cufflinks software was utilized to splice these mapped reads based on the reference genome sequence [21]. DEGs were screened using DESeq software [22] under the following standard parameters: FDR < 0.05 and log2|FC| ≥ 1. To research the function of DEGs, multiple bioinformatic tools were utilized to analyze the annotation, classification, and metabolic pathway. The DEGs were conducted to perform GO enrichment analysis based on Gene Ontology (The Gene Ontology Resource: 20 years and still GOing strong 2019) [23]. The DEGs based on KEGG [24] and KOBAS [25] were used to identify the enriched metabolic pathways or signal transduction pathways. KOBAS software was used to test the enrichment statistics in the KEGG pathway.

#### 2.1.4. Data Analysis

All values are expressed as the mean ± standard deviation (SD). GraphPad Prism 6 was used to analyze the data using a one-way analysis of variance (ANOVA) followed by Tukey's test.

## 3. Results

### 3.1. Physiological Changes of Maize in Response to Drought Stress

In our research, the contents of soluble saccharide, POD, SOD, CAT, MDA, and protein were measured in maize roots at polyethylene glycol- (PEG-) induced drought stress (5%, 10%, 15%, and 20%) (Figure 1(b)). With the PEG concentration increased, the content of saccharide, SOD, CAT, and MDA showed an upward trend. On the contrary, the content of Pro showed a downward trend as the PEG concentration increased. At the same time, we found that the content of POD showed an increase at first and then decrease, reaching maximum at 15% polyethylene glycol- (PEG-) induced drought stress.

### 3.2. Transcriptomic Analysis of Maize Responses to Drought Stress

An overview of the RNA-Seq reads derived from the sequencing results is listed in Supplemental Table 1. 100.95 GB clean data were obtained from the 15 samples. The average Q30 and GC content values of these clean reads were greater than 93.55% and 52.94%, respectively, indicating that the data were reliable and available for subsequent analysis.

**Figure 2:**
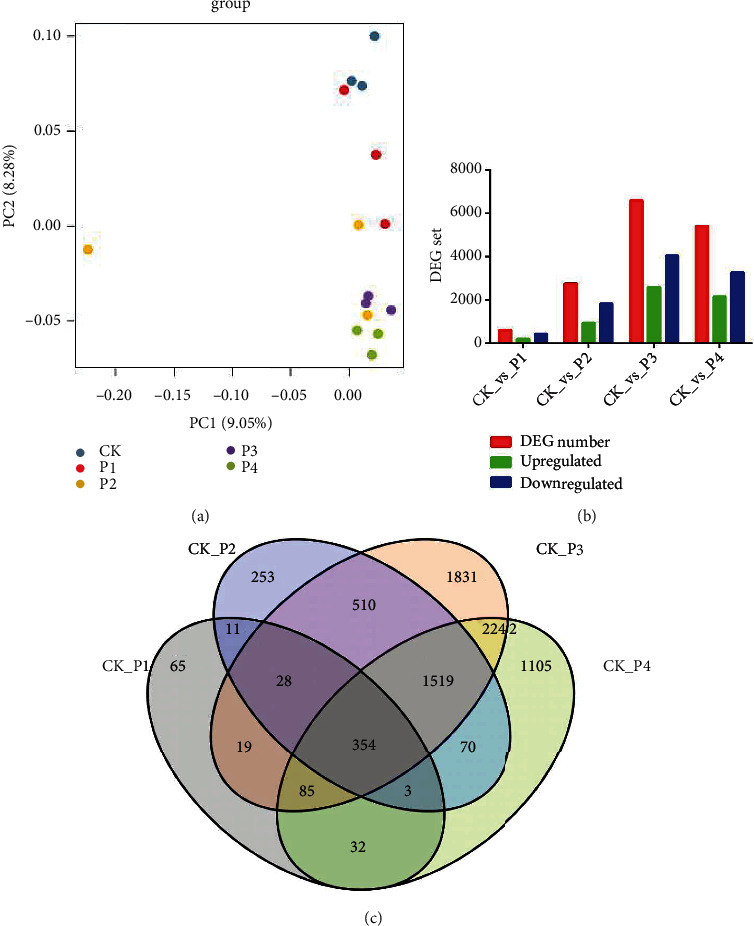
DEGs analysis. (a) PCA plot of PEG-induced drought stress in maize. (b) Expression patterns of the DEGs. (c) The Venn diagram showing the comparison of DEGs expressed at each comparison group.

To identify the global transcriptomic changes induced by drought stress, an analysis of transcriptome data was conducted. Principal component analysis (PCA) was utilized to assess gene expression levels for each replicate (Figure 2(a)). Samples from 5% PEG, 10% PEG, 15% PEG, and 20% PEG clustered far from the 0% PEG (control) samples, indicating that drought stress induced differential expression of gene.

To determine the DEGs involved in response to drought stress, upregulated DEGs and downregulated DEGs were screened with a threshold of |log2 (FC)| ≥ 1 and *p* value ≤ 0.05. Compared with the control group, there were 597 (189 upregulated and 408 downregulated), 2748 (927 upregulated and 1821 downregulated), and 6588 (2561 upregulated and 4027 downregulated) genes that showed different levels of expression after 5% PEG, 10% PEG, 15% PEG, and 20% PEG drought treatment, respectively (Figure 2(b)). Meanwhile, the distribution of DEGs at the four comparisons was calculated and presented in a Venn diagram, and 354 common DEGs (Figure 2(c)) were identified in these comparisons.

### 3.3. KEGG Enrichment Analysis

**Figure 3:**
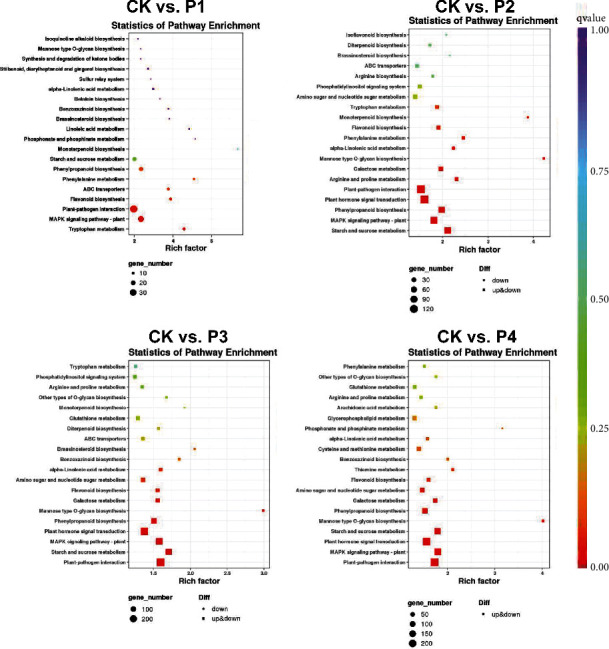
KEGG pathway enrichment analysis of the DEGs after 5% PEG, 10% PEG, 15% PEG, and 20% PEG drought treatment, respectively. The experimental comparisons were based on the hypergeometric test, while the significance of the enrichment of the KEGG pathway is based on the *q* value (*q* < 0.05). The “rich factor” shows the DEG ratio to the total gene number in specific pathways.

Simultaneously, KEGG enrichment analysis was conducted to explore the function of the DEGs (Figure 3). Interestingly, MAPK signaling pathway and starch and sucrose metabolism pathways are enriched in 5%, 10%, 15%, and 20% PEG treatment groups. Phytohormone signal transduction is enriched in 10%, 15%, and 20% PEG treatment groups. At 5% PEG drought treatment, the DEGs were also significantly enriched into taurine and hypotaurine metabolism, plant-pathogen interaction, flavonoid biosynthesis, and ABC transports. At 10% PEG drought treatment, phenylpropanoid biosynthesis, plant-pathogen interaction, galactose metabolism, arginine, and proline metabolism were also significantly enriched.

Interestingly, at 15% and 20% PEG drought treatment, enrichment pathways are also involved in mannose type O-glycan biosynthesis and phenylpropanoid biosynthesis. Furthermore, the 354 common DEGs performed KEGG enrichment analysis, indicating that flavonoid biosynthesis, mannose type O-glycan biosynthesis, and benzoxazinoid biosynthesis were significantly enriched.

### 3.4. Expression of Transcription Factors under Drought Stress

**Figure 4:**
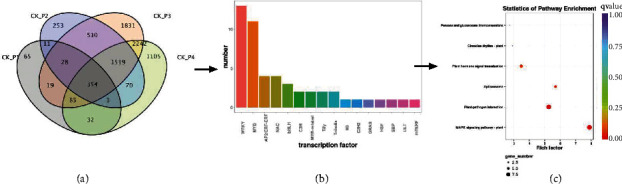
Screened transcription factor analyzed from 354 common DEGs. (a) The Venn diagram showing the comparison of DEGs expressed at each comparison group. (b) The number of transcription factor. (c) The function of transcription factor.

The 50 TFs, categorized into 15 categories, were screened from the 354 common DEGs during drought stress (Figure 4(a)). The largest group of TFs was the WRKY family, followed by the MYB, whereas other TFs belonged to the AP2/ERF-ERF, NAC, bHLH, and C3H (Figure 4(b)). In 5%, 10%, 15%, and 20% PEG treatment group, 7 TFs, including Zm00014a022627 (B3 DNA-binding domain), Zm00014a028890 (HSF), Zm00014a017550 (SBP), Zm00014a006206 (bHLH), Zm00014a037517 (Myb), Zm00014a002630 (Myb), and Zm00014a015163 (CCCH), were evidently high expression. Meanwhile, other 43 TFs were downregulated in 5%, 10%, 15%, and 20% PEG treatment groups (Supplemental Table 2). Then, KEGG enrichment analysis was conducted to explore the function of TFs. MAPK signaling pathway, hormone signal transduction, plant-pathogen interaction, and spliceosome were significantly enriched (Figure 4(c)).

### 3.5. Expressional Regulation in MAPK Signaling Pathway in Response to Drought Stress

**Figure 5:**
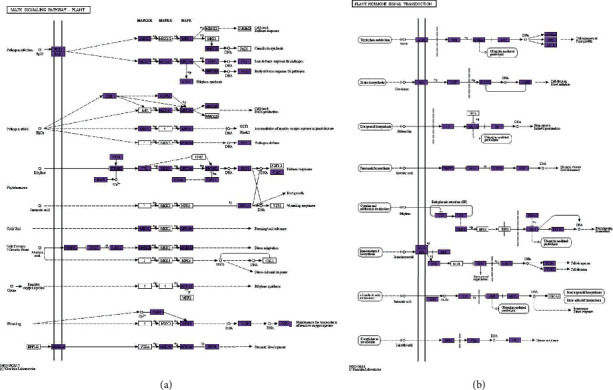
KEGG map of MAPK signaling pathway (a) and hormone signal transduction (b). It is an analysis of DEGs, comparing drought-treated and control samples. Boxes in a color frame indicate that the corresponding DEGs were down- or upregulated in the drought-treated samples, and the boxes with a white frame suggest that the expression levels of the related genes were not significantly changed in our RNA-Seq.

216 DEGs were found to participate in the MAPK signaling pathway in response to drought stress (Figure 5(a)). 27 genes were differentially expressed under drought stress in 5% PEG, including 22 downregulated genes and 5 upregulated genes, in which transcription factor bHLH94 and transcription factor bHLH96 were upregulated.

There were 20 upregulated genes and 72 downregulated genes in MAPK signaling pathway between the CK and 10% PEG drought stress group. Meanwhile, transcription factors bHLH35 and bHLH96 were overexpressed in 10% PEG drought stress group. 186 genes, including 46 upregulated and 140 downregulated genes, were differentially expressed under 15% drought compared with those of the CK. Transcription factors bHLH96 and bHLH35 were upregulated. Compared with CK, 116 downregulated genes and 53 upregulated genes were screened in 20% PEG drought stress in this pathway. Besides, two transcription factors bHLH (bHLH35 and bHLH96) displayed an upregulated expression in the MAPK signal transduction pathway.

### 3.6. Expressional Regulation in Hormone Signal Transduction Network in Response to Drought

Since DEGs enriched hormone signal transduction pathways, we further investigated the regulation of these DEGs and KEGG pathway (Figure 5(b)). A total of 302 DEGs were identified, including auxin, brassinosteroid, and ethylene. In CK vs. 5% PEG group, 14 downregulated genes and 8 upregulated genes were found. With the aggravation of drought stress, the number of down- and upregulated genes in hormone signal transduction network was increased. Compared with CK, 85, 171, and 139 genes were downregulated, while 38, 74, and 82 genes were upregulated in 10%, 15%, and 20% PEG.

Auxin-responsive protein IAA9, ethylene response sensor 2, auxin response factor 10, and auxin-responsive protein IAA1 were over-regulated in response to 10% PEG drought compared with CK. 5 auxin genes (auxin response factor 10, auxin-responsive protein IAA9, auxin response factor 14, auxin-responsive protein IAA1, and auxin-responsive protein IAA27) and 1 ethylene response sensor 2 were upregulated in response to 15% PEG drought compared with CK. In CK vs. 20% PEG group, brassinosteroid LRR receptor kinase BRL2, brassinosteroid LRR receptor kinase BRL3, ethylene receptor 3, auxin-responsive protein IAA7, and auxin response factor 17 were upregulated. It indicated that the genes related to hormone signal transduction play an important function in drought stress.

## 4. Discussion

Drought stress often exhibits adverse effects and brings out cellular damage. It is essential to explore the expression profile of drought-responsive genes to realize the molecular mechanisms associated with drought stress tolerance in crops [26]. In this study, PEG was employed in the media to create rapid drought stress through water deprivation, which was consistent with the previous research [27].

Our research found that the contents of saccharide, SOD, CAT, and MDA are significantly increased with the enhancement in drought intensity. In response to drought stress, the dramatic increase in ROS levels in plants leads to severe oxidative damage to DNA, proteins, and lipids [28]. These reactive oxygen species, such as H_2_O_2_, directly attack membrane lipids and increase lipid peroxidation. MDA is a marker for membrane lipid peroxidation, whose level can indicate oxidative damage [29]. It is reported that SOD and CAT played major roles in the defense against toxic ROS. SOD and CAT were increased in the early phase of drought and reduced as the drought worsened [10]. It explained why the content of SOD, CAT, and MDA showed an upward trend with the increase in PEG concentration.

Transcriptional reprogramming is one of the major mechanisms that plants undergo during stress tolerance. In cassava, DEGs showed significant changes when the PEG stress period was enhanced from 3 h to 24 h. During drought stress, plants undergo significant changes in gene expression by regulating cellular processes in order to survive under drought conditions [2]. Similar results were found in our results, and there were 597, 2748, and 6588 genes which were differentially expressed after 5% PEG, 10% PEG, 15% PEG, and 20% PEG drought treatment, respectively. Meanwhile, the number of DEGs evidently increased with aggravation of drought. The elucidation of drought resistance in plants through different gene expression approaches provides valuable information to identify probable drought resistance mechanisms.

The transcription factors, as regulatory proteins, exert vital functions in drought tolerance by synchronizing the signaling network and gene expression under stress. Some of the most critical players in the abscisic acid pathway are drought-responsive element binding (DREB) proteins that are a part of AP2/ethylene response factor transcription factors that bind to promoters of some family genes needed to be expressed under abiotic stresses [30]. The drought tolerance of NAC TFs has been demonstrated in various crops, such as rice [31] and soybean [32]. Yang et al. found 13 WRKY genes involved in drought response in the study of weeping forsythia, most of which responded to drought stress by regulating the abscisic acid signaling pathway [33]. *GmERF113* downregulates abscisic acid 8′-hydroxylase and upregulates various drought-related genes, which improves drought resistance and affects the ABA content in soybean [34]. Overexpression of SlbHLH96 in tomatoes improves drought tolerance by stimulating the expression of genes encoding antioxidants, ABA signaling molecules, and stress-related proteins [35]. Other drought-responsive TFs revealed differential expression under PEG-induced drought stress [36]. Consistent with the above research, in our dataset, the expression of 50 transcription factor genes, classified into 15 families, was significantly different under PEG stress, compared to the control (Figure 4(b)). The 50 transcription factor genes were involved in 7 upregulated genes and 43 downregulated genes. Our results have long exhibited distinct expression profiles of TFs in different plants under drought stress. More DETFs suggested the role of a more complex transcriptional regulation network and improved the drought resistance of maize.

The starch and sucrose metabolism was significantly enriched, which was found in Sophora moorcroftiana [37] and Vicia sativa [38] exposed to drought stress. Our results were consistent with the transcriptome data above. The KEGG analysis showed that MAPK signaling pathway-plant, phytohormone signal transduction, and starch and sucrose metabolism were significantly enriched, which means that maize consumed much energy when suffering drought stress.

The MAPK cascade is a multigene family of regulatory networks used to deliver intracellular and extracellular signals to the nucleus, allowing the cell to adjust appropriately to stimulation [39]. In the present study, 216 DEGs were mapped to the MAPK pathway, including the expression of MAPKKK, MAPKK, and MAPK. Evidently, the down- and upregulated genes were increasing with aggravation of drought stress from 5% to 20% PEG. Phytohormones exerted significant functions in response to drought stress. Drought induction can induce the secretion of phytohormones that mediate the immediate cellular responses by triggering phytohormone signaling pathways [40]. In our research, 302 DEGs were found, including auxin, brassinosteroid, and ethylene. Ethylene, a plant-growth regulator, regulates the growth of plants by undertaking various developmental changes in the plant under drought conditions [41]. Ethylene response sensor 2 was upregulated under 5%, 10%, 15%, and 20% PEG stress, suggesting that an ethylene-induced defense mechanism may be activated under PEG-induced stress conditions. Previous studies indicated that auxin could mediate the expression of auxin responding genes, and the ARF family mediated the roles of IAA during plant growth [42, 43].

**Figure 6:**
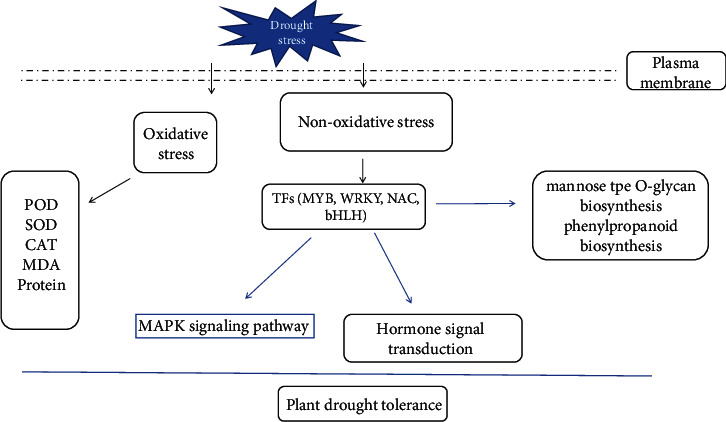
A putative regulation mechanisms of drought stress.

Studies illuminated that IAA is associated with drought tolerance in plants, and wild Arabidopsis plants pretreated with IAA exhibit improved drought resistance. Transcriptome data indicated that exogenous IAA and drought induced rice AUX/IAA genes. AUX/IAA1 was also upregulated in sorghum due to drought [44]. The findings explained why the 5 IAA genes (auxin response factor 10, auxin-responsive protein IAA9, auxin response factor 14, auxin-responsive protein IAA1, and auxin-responsive protein IAA27) were upregulated in PEG stress. Especially, the two TFs, including bHLH 35 and bHLH 96, involved in MAPK signal pathway and plant hormone pathway are significantly upregulated in 5%, 10%, 15%, and 20% PEG stress groups. MfbHLH38 enhanced tolerance to drought in Arabidopsis through increasing water retention ability, regulating osmotic balance, and possibly participating in ABA-dependent stress-responding pathway [45]. PebHLH35 functions as a positive regulator of drought stress responses by regulating stomatal density, stomatal aperture, photosynthesis, and growth [46]. As shown in Figure 6, the regulation mechanisms of drought stress are related to multiple pathways in maize roots. The mechanism of drought tolerance correlates with the MAPK signal pathway and hormone signal transduction, which were regulated by many TFs, including MYB, WRKY, NAC, and bHLH. In short, many key genes including transcription factors, hormone signal transduction-related genes, and MAPK signaling pathway-related genes were identified and interacted to improve drought tolerance in maize.

## 5. Conclusion

In this study, at normal conditions and polyethylene glycol- (PEG-) induced drought stress (5%, 10%, 15%, and 20%) in the root during the seedling stage, the physiological indexes showed that maize responded rapidly to drought stress. Transcriptome analysis indicated that the numbers of DEGs gradually increased with the aggravation of drought stress. These DEGs may be strongly related to drought tolerance in maize. Meanwhile, our research may provide a theoretical basis for enhancing drought resistance in other plants.

## Data Availability

Data to support the findings of this study is available on reasonable request from the corresponding author.

## Supplementary Materials

Supplementary 1 Supplemental Table 1: an overview of the RNA-Seq reads derived from the sequencing results.

Supplementary 2 Supplemental Table 2: forty-three transcription factors were downregulated in 5%, 10%, 15%, and 20% PEG treatment groups.

Supplementary 3 Supplemental Table 3: a collection of 216 DEGs in the MAPK signaling pathway in response to drought stress.

Supplementary 4 Supplemental Table 4: 302 DEGs in hormone signal transduction network in response to drought.
