# Supplementary material for: Physiological Characteristic Changes and Transcriptome Analysis of Maize (Zea mays L.) Roots under Drought Stress
Source: Int J Genomics. 2024 Jan 17;2024:5681174. doi: 10.1155/2024/5681174 (PMC10807950; doi:10.1155/2024/5681174)
Supplement: Supplementary 1 — Supplemental Table 1: an overview of the RNA-Seq reads derived from the sequencing results. [file 5681174.f1.docx]

Supplemental Table 1 Statistical table of sequencing data

| Samples | Clean reads | Clean bases | GC Content (%) | ≥Q30 |
| --- | --- | --- | --- | --- |
| CK-1 | 25,076,401 | 7,501,801,952 | 54.44% | 94.31% |
| CK-2 | 25,372,562 | 7,588,278,858 | 54.85% | 94.42% |
| CK-3 | 25,201,181 | 7,542,468,436 | 53.71% | 93.91% |
| P1-1 | 20,742,185 | 6,208,533,288 | 54.89% | 93.70% |
| P1-2 | 21,433,987 | 6,416,738,524 | 54.08% | 93.94% |
| P1-3 | 19,439,144 | 5,809,942,262 | 53.66% | 94.13% |
| P2-1 | 23,604,545 | 7,067,740,396 | 54.14% | 93.55% |
| P2-2 | 26,842,254 | 8,031,606,470 | 53.50% | 93.56% |
| P2-3 | 20,860,411 | 6,239,168,042 | 52.87% | 93.97% |
| P3-1 | 25,261,275 | 7,556,986,270 | 53.22% | 93.85% |
| P3-2 | 19,493,621 | 5,831,824,220 | 52.94% | 93.74% |
| P3-3 | 24,004,243 | 7,167,141,472 | 53.77% | 93.83% |
| P4-1 | 19,398,817 | 5,804,949,160 | 53.26% | 94.03% |
| P4-2 | 19,781,059 | 5,916,694,096 | 53.02% | 93.75% |
| P4-3 | 20,949,856 | 6,264,048,084 | 53.65% | 93.75% |

Note: (1) Samples: analysis numbers; (2) Clean reads: the total number of pair-end Reads in Clean Data; (3) Clean bases: total number of Clean Data; (4) GC content: Clean DataGC content, that is, the percentage of G and C bases in the total bases in Clean Data; (5) ≥Q30%: Clean Data is the percentage of bases with mass value greater than or equal to 30.
